# Microenvironment‐Based Diabetic Foot Ulcer Nanomedicine

**DOI:** 10.1002/advs.202203308

**Published:** 2022-11-24

**Authors:** Fang Huang, Xiangyu Lu, Yan Yang, Yushan Yang, Yongyong Li, Le Kuai, Bin Li, Haiqing Dong, Jianlin Shi

**Affiliations:** ^1^ Key Laboratory of Spine and Spinal Cord Injury Repair and Regeneration Ministry of Education Tongji Hospital School of Medicine Tongji University 389 Xincun Road Shanghai 200065 China; ^2^ Shanghai Tenth People's Hospital Shanghai Frontiers Science Center of Nanocatalytic Medicine The Institute for Biomedical Engineering and Nano Science School of Medicine Tongji University Shanghai 200092 China; ^3^ State Key Laboratory of High Performance Ceramics and Superfine Microstructure Shanghai Institute of Ceramics Chinese Academy of Sciences; Research Unit of Nanocatalytic Medicine in Specific Therapy for Serious Disease Chinese Academy of Medical Sciences (2021RU012) Shanghai 200050 China; ^4^ Shanghai Skin Disease Hospital School of Medicine Tongji University Shanghai 200443 China; ^5^ Department of Dermatology Yueyang Hospital of Integrated Traditional Chinese and Western Medicine Shanghai University of Traditional Chinese Medicine Shanghai 200437 China

**Keywords:** diabetic foot ulcers, microenvironment, nanomedicine, therapeutic strategy, wound healing

## Abstract

Diabetic foot ulcers (DFU), one of the most serious complications of diabetes, are essentially chronic, nonhealing wounds caused by diabetic neuropathy, vascular disease, and bacterial infection. Given its pathogenesis, the DFU microenvironment is rather complicated and characterized by hyperglycemia, ischemia, hypoxia, hyperinflammation, and persistent infection. However, the current clinical therapies for DFU are dissatisfactory, which drives researchers to turn attention to advanced nanotechnology to address DFU therapeutic bottlenecks. In the last decade, a large number of multifunctional nanosystems based on the microenvironment of DFU have been developed with positive effects in DFU therapy, forming a novel concept of “DFU nanomedicine”. However, a systematic overview of DFU nanomedicine is still unavailable in the literature. This review summarizes the microenvironmental characteristics of DFU, presents the main progress of wound healing, and summaries the state‐of‐the‐art therapeutic strategies for DFU. Furthermore, the main challenges and future perspectives in this field are discussed and prospected, aiming to fuel and foster the development of DFU nanomedicines successfully.

## Graphical abstract

The definition of “diabetic foot ulcer (DFU) nanomedicine” is first introduced to bridge “DFU” and “nanomaterials”. Then, this review's focus is be placed on the normal wound healing process, the complicated microenvironment of DFU, and the construction and treatment strategies of various nanosystems in “DFU nanomedicine”. Last, the opportunities and challenges of microenvironment‐based “DFU nanomedicine” are discussed to drive its development.

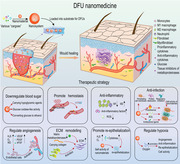

## 1 Introduction

Diabetic foot ulcers (DFU) have imposed severe health and economic burden globally. Approximately 19%–34% of diabetics are influenced by DFU and up to one‐third of all diabetes care costs are estimated to be for lower‐limb‐related problems (about $5.9 billion annually in the United States).^[^ ^1^ ^]^ The recurrence rate within one year is ≈40% after successful healing, making DFU treatment extremely challenging.^[^ ^2^ ^]^ It is generally accepted that persistent hyperglycemia caused diabetic neuropathy and vasculopathy are the main aetiologies of DFU, and further bacterial infection exacerbates the difficulty of treating DFU.^[^ ^3^ ^]^ In brief, hyperglycemia upregulates the expression of proinflammatory factors, causing oxidative stress on nerve cells and neuropathy (mainly nerve damage in the foot).^[^ ^4^ ^]^ Specifically, motor neuropathy may lead to muscle deformity and atrophy, and autonomic neuropathy is prone to cause skin dehiscence. Worse still, lesions of widespread sensory nerves in the skin of diabetics reduce or lose basic “sensation” and “pain,” resulting in the insensitivity or even unconscious of the nervous system to the wound and consequently greatly increased risk of ulcers and even amputation.^[^ ^3^, ^5^ ^]^ In addition, hyperglycemia causes glycation of hemoglobin, narrowing of blood vessels, and alteration of the erythrocyte membrane, leading to inadequate oxygen supply and vasculopathy to the wound tissue.^[^ ^6^ ^]^ Vasculopathy may also cause or intensify the loss of “sensation” in patients. On these bases, combined bacterial infections and biofilm formation further complicate the wound microenvironment. Finally, the various predisposing factors discussed above prevent diabetic wounds from undergoing four stages of normal wound healing: hemostasis, inflammation, proliferation, and remodeling. Instead, the wound may stagnate in the long‐term inflammatory phase, leading to the formation of nonhealing chronic wounds, i.e., DFU.^[^ ^7^ ^]^ For more details on cellular and molecular events involved in DFU, one can refer to comprehensive reviews.^[^ ^6^, ^8^ ^]^

Complicated pathogenesis of DFU results in a complex microenvironment, such as hyperglycemia, ischemia, hypoxia, hyperinflammation, persistent infection, high protease activity,^[^ ^9^ ^]^ inadequate energy supply,^[^ ^10^ ^]^ local necrotic tissue,^[^ ^11^ ^]^ and wide range variation of pH (7.0–8.9) in chronic phase.^[^ ^12^ ^]^ Based on the characteristics of DFU, the standard clinical treatment includes surgical debridement, pressure off‐loading, antibiotic therapy, and wound dressing.^[^ ^13^ ^]^ Unfortunately, long‐term and repeated debridement will bring extreme pain and economic burdens to the patients. Furthermore, the emergence of drug‐resistant microorganisms may invalidate the effect of antibiotics; meanwhile, conventional dressings are monofunctional and necessitate frequent changes.^[^ ^14^ ^]^ Therefore, adjuvant therapies such as gene/growth factor therapy and stem cell therapy have gradually attracted clinical attention. In particular, stem cells are crucial for the wound healing process. As a pivotal “seed cell,” stem cells recruit macrophages and endothelial lineage cells to ischemic and damaged tissue, secrete growth factors and establish a favorable environment for wound repair.^[^ ^15^ ^]^ Nevertheless, several issues regarding effective delivery remain, such as 1) the short half‐life of therapeutic genes/growth factors; 2) the highly complicated DFU microenvironment with extensive proteases; and 3) the short survival time of stem cells in DFU microenvironment results in disoptimal retention in precise positions.^[^ ^15^, ^16^ ^]^

**Figure 1:**
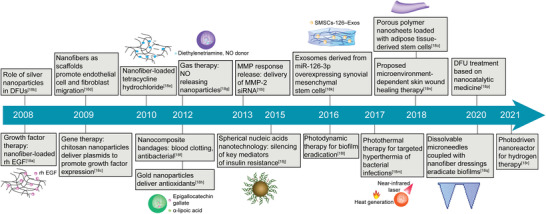
Timeline of progress in “diabetic foot ulcer (DFU) nanomedicine”.^[^ ^18^ ^]^

Fortunately, nanotechnology possesses advantages such as drug delivery and controlled release that can address the above issues efficiently. More encouragingly, except for the role of “deliverers,” a number of nanomaterials also act as “healers” in the treatment of DFU, in which the nanomaterials themselves are capable of treating diseases directly. Over the past decade, novel strategies based on nanosystems for remodeling the DFU microenvironment have been rapidly developed (**Figure** **1**), forming a concept of “DFU nanomedicine,” i.e., developing nanosystems for DFU therapy. Nanotechnology‐based DFU therapies are well documented and summarized by focusing on the types of nanocarriers and drugs.^[^ ^17^ ^]^ Herein, the microenvironment‐guided design motifs for nanomedicine, which, however, is of great significance in developing nanosystems for greatly enhanced effectiveness in DFU clinical treatment, will be covered. In this review, the main processes of wound healing, the microenvironment of DFU, and nanotechnology‐based strategies to remodel DFU's microenvironment will be summarized to provide constructive ideas and implications for developing more effective DFU nanomedicines.

## 2 Main Processes of Wound Healing

Unsurprisingly, the skin is susceptible to damage from various external/internal factors. Once the wound occurs, the typical external appearance is bleeding, accompanied by microbial infection which is not beneficial for health. Thus, effective wound healing is of critical importance, and it generally contains following four continuous and overlapping events.

### 2.1 Hemostasis

Hemostasis is the first response of the body to a wound. Immediately after injury, blood vessels constrict to reduce bleeding; subsequently, platelets accumulate in the wound and activate, forming the platelet plug, a process known as primary hemostasis.^[^ ^19^ ^]^ Activated platelets also secrete cytokines such as platelet‐derived growth factor (PDGF), transforming growth factor‐*β* (TGF‐*β*), and epidermal growth factor (EGF) to recruit inflammatory cells and promote the proliferation and migration of fibroblasts and keratinocytes. Next, to enable platelet plug clotting and strengthening, the classical endogenous and exogenous coagulation pathways are initiated, stimulating the formation of fibrin, which in turn forms fibrin chains in the wound. Then, under the action of Factor XIII, the fibrin chains interconnect, establishing a dense fibrin network that eventually forms a thrombus at the wound. The thrombus serves as a temporary substrate for wound healing, provides a scaffold for cell migration, and protects the wound from pathogenic invasion.^[^ ^8^, ^20^ ^]^

### 2.2 Inflammation

The hallmark of the onset of the inflammatory phase is the recruitment of inflammatory cells, which overlaps with the hemostatic phase.^[^ ^21^ ^]^ The recruitment of inflammatory cells is associated with the release of various “danger signals” at the wound site, such as pathogen‐associated molecular patterns (PAMPs) and danger‐associated molecular patterns (DMEMs).^[^ ^8^, ^22^ ^]^ Neutrophils are the “first responders” to the signal,^[^ ^23^ ^]^ followed by monocytes, macrophages, lymphocytes, and others that arrive at the site of injury. In acute wounds, neutrophils fight against microorganisms through phagocytosis and subsequent releasing of reactive oxygen species (ROS), they also express matrix metalloproteinases (MMPs) to clear necrotic tissue, and secrete cytokines to amplify inflammatory signals; in addition, neutrophils release vascular endothelial growth factor (VEGF) in preparation for the proliferative phase.^[^ ^23^, ^24^ ^]^ Finally, neutrophils are cleared by phagocytosis of macrophages or re‐entry into the vascular system, which means inflammation subsides. For macrophages, the origin of macrophages at the wound site is related to monocyte differentiation and local tissue residence. In early inflammation, proinflammatory macrophages (M1 types) produce ROS and proinflammatory cytokines (e.g., interleukin‐6 [IL‐6], tumor necrosis factor‐*α* [TNF‐*α*], IL‐1) to exert bactericidal effects; subsequently, M1 macrophages remove neutrophils in time through “efferocytosis.” Finally, driven by “cytokinesis” or other signals, M1 macrophages polarize to the M2 type and the wound gradually transitions to the proliferative phase.^[^ ^19^, ^21^ ^]^ The polarization of macrophages is crucial for subsequent wound healing.

### 2.3 Proliferation

The migration of fibroblasts is the landmark event for the arrival of the proliferative phase. During the proliferative phase, the establishment of granulation, angiogenesis, and re‐epithelialization can be observed simultaneously.^[^ ^8^, ^24^, ^25^ ^]^

Granulation formation is closely related to fibroblast activity. Fibroblasts at the wound originate from the proliferation and migration of fibroblasts at the wound margin, transdifferentiation of M2 macrophages, and differentiation of circulating fibroblasts.^[^ ^26^ ^]^ Stimulated by cytokines released from platelets, macrophages, and basic fibroblasts, fibroblasts migrate to the injured site, proliferate and secrete proteases and extracellular matrix (ECM) components to construct granulation tissue.^[^ ^8^, ^19^ ^]^ At this stage, granulation tissue will replace the thrombus as a scaffold for new cells (e.g., macrophages) or other components (e.g., new blood vessels).^[^ ^8^, ^25^ ^]^

Angiogenesis is another important behavior in this phase. Stimulated by moderate hypoxia, cytokines, and protein hydrolases, endothelial cells are activated to induce angiogenesis.^[^ ^8^, ^24^, ^27^ ^]^ Specifically, under the action of enzymes, endothelial cells “escape” from damaged vessels, proliferate and migrate toward proangiogenic signals to build new vascular networks. In addition to endothelial cells, others (macrophages, pericytes, and smooth muscle cells) are also involved in this process. M2 macrophages express cytokines such as VEGF and PDGF to stimulate angiogenesis; pericytes and smooth muscle cells stabilize the neovascularization.^[^ ^27^ ^]^

Re‐epithelialization is mainly associated with the migration and proliferation of keratinocytes at the wound margin, which is stimulated by cytokines secreted by various cells. In turn, keratinocytes can generate signals that can act on other cells to promote macrophage activation, granulation formation, angiogenesis, and so on.^[^ ^8^, ^10^ ^]^

### 2.4 Remodeling

The remodeling stage is the final stage of wound healing, with the primary objectives of restoring tissue structure and increasing tensile strength.^[^ ^28^ ^]^ In this stage, type III collagen in granulation tissue is gradually replaced by type I collagen. In addition, under the action of protease and its inhibitor, ECM in granulation tissue undergoes rearrangement and positioning, which can change its haphazard distribution to increase the tensile strength of the tissue.^[^ ^29^ ^]^ Furthermore, driven by signals such as *α*‐smooth muscle actin (*α*‐SMA) and growth factors, some fibroblasts differentiate into myofibroblasts to promote matrix contraction.^[^ ^30^ ^]^ Finally, most of the macrophages, myofibroblasts, and endothelial cells involved in wound repair disappear through apoptosis, differentiation, or other unknown mechanisms.^[^ ^19^, ^28^ ^]^ The duration of remodeling is determined by the extent of the injury and can last from weeks to years.

There is no doubt that wound healing involves the crosstalk of cell–cell, cell–factor, cell–tissue, cell–vascular systems, etc., and each healing process will provide the corresponding signal for the next stage.^[^ ^9^, ^11^ ^]^ Surprisingly, the organisms can protect “themselves” through such complex but near‐perfect work. The investigation of these exact mechanisms relies on the efforts of many scientists, but there are still some unclear mechanisms, which will be clearly investigated in the future.

## 3 DFU Wounds and the Microenvironment of Perturbed Wound Healing

**Figure 2:**
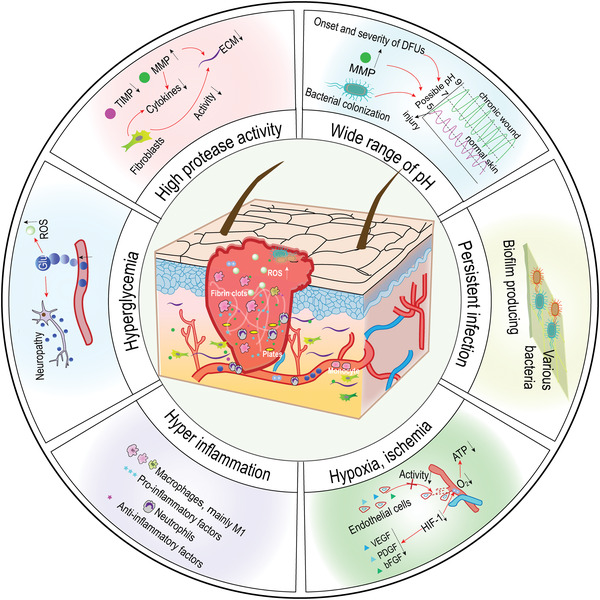
The complex microenvironment of DFU, including hyperglycemia, hyperinflammation, hypoxia, etc., renders the wound nonhealing.

Distinct from the acute wound described above, DFU is a chronic wound with chronic persistent inflammation and the healing period generally lasted for more than four weeks in clinic. Although the healing process of DFU wounds is similar to normal acute wound, they stagnate at some stage.^[^ ^31^ ^]^ Apparent differences as tabulated in **Table** **1** between acute and DFU wounds. In general, the sophisticated microenvironment perturbed the cascade healing of DFU wounds. The DFU microenvironment, in short, refers to the extracellular compartment where the cells and signals (e.g., growth factors) involved in the DFU development or healing process. It is composed of both internal and external microenvironment components.^[^ ^32^ ^]^ However, the discussion of microenvironmentally guided nanostrategies in DFU healing within the literature is usually piecemeal. Therefore, in this section, we will mainly discuss the DFU microenvironment involved in the application of nanomedicines, such as hyperinflammation, hyperproteases, and hypoxia (**Figure** **2**), as well as how the microenvironment prevents healing events.

**Table 1:** Differences between acute wouds and DFU wounds

Wound stage	Acute wounds	DFU wounds
Hemostasis	**Quick**	**Quick**
	(1) Due to the reflex contraction of the vascular smooth muscle and the release of vasoconstrictors from the damaged endothelial cells, hemostasis is initiated immediately after injury;^[^ ^8a^ ^]^ (2) Platelets are the main regulatory cells.	(1) Unrelated to massive blood loss, some wounds are accompanied by slight blood loss; hemostasis is initiated immediately after trauma;^[^ ^33^ ^]^ (2) Wounds mostly manifest as ulceration and accumulation of necrotic debris at the edges.^[^ ^31^ ^]^
Inflammation	**Transient**	**Chronic and persistent**
	(1) Transient immune response;^[^ ^34^ ^]^ (2) Transformation of macrophage phenotype facilitates tissue repair;^[^ ^35^ ^]^ (3) Low bacterial load.	(1) Massive immune cell infiltration and chronic ongoing immune response;^[^ ^34^ ^]^ (2) The hyperglycemic environment induces the pro‐inflammatory phenotype of macrophages^[^ ^36^ ^]^ (3) More susceptible to infection (immune cells may have reduced bactericidal and phagocytic activity).^[^ ^31^ ^]^
Proliferation	**Easy**	**Difficult**
	(1) Fibroblast activation; (2) Neovascularization to alleviate hypoxia and ischemia; (3) Controlled MMP activation;^[^ ^37^ ^]^ (4) Keratinocyte activation.	(1) Fibroblast senescence, unresponsive to migratory stimulant TGF‐*β*;^[^ ^34^ ^]^ (2) Little vascular sprouting, inadequate oxygen and nutrient supply, and reduced expression of HIF‐1*α* and HIF‐1 target genes;^[^ ^31^, ^35^ ^]^ (3) Microenvironment induces increased MMP expression;^[^ ^36^ ^]^ (4)Re‐epithelialization is stagnant.^[^ ^31^ ^]^
Remodeling	**Easy**	**Difficult**
	(1) ECM remodeling; (2) The remodeling phase lasts for months or even years, depending on the condition of the wound.	(1) ECM decomposition; (2) Remodeling stagnation.

### 3.1 Hyperglycemia

Hyperglycemia is one of the microenvironment characteristics of DFU and is related to insulin disturbance (deficiency or resistance). Under normal conditions, intracellular glucose is rarely metabolized by the polyol pathway. However, under the stimulation of hyperglycemia, the glucose metabolized via the polyol pathway significantly increases (more than 30%), accompanied by activation of the hexosamine pathway and protein kinase C pathway.^[^ ^38^ ^]^

Such abnormal glucose metabolism inhibits the expression of antioxidants while inducing advanced glycation end products and promoting oxidative stress, which further aggregates inflammation.^[^ ^4^, ^6^, ^39^ ^]^ Additionally, hyperglycemia also increases the risk of bacterial infection, hindering inflammation from subsiding.^[^ ^6^ ^]^

In parallel, local hyperglycemia can induce the apoptosis of endothelial cells, suppress the migratory capacity of fibroblasts and keratinocytes, and promote significant expression of MMP‐1, thereby limiting the process of proliferation and remodeling.^[^ ^13^, ^40^ ^]^

### 3.2 Hyperinflammation

Hyperinflammation in DFU is associated with an imbalance of redox homeostasis induced by hyperglycemia as previously discussed. Specifically, during the inflammatory phase, hyperglycemia hampers chemokine expression, which delays the entry of monocytes and macrophages into the wound site, preventing the timely clearance of neutrophils, leading to an increase in oxidative stress in the wound. In addition, hyperglycemia‐induced epigenetic alterations, involving histone methylation and acetylation, upregulate the expression of pro‐inflammatory factors (e.g., TNF‐*α*, IL‐1, IL‐6) and facilitate M1 macrophage polarization. The presence of M1 macrophages further upregulates proinflammatory factors, leading to a vicious cycle of M1 macrophage infiltration and oxidative stress, which are primary factors in the persistence of inflammation.^[^ ^41^ ^]^

Furthermore, persistent oxidative stress facilitates the senescence of fibroblasts, endothelial cells, keratinocytes, and mesenchymal stem cells, which are extremely detrimental to the healing process including the formation of granulation tissue, blood vessels, and epithelium.^[^ ^39^, ^41^, ^42^ ^]^ In addition, prolonged inflammation is paralleled by collagen damage and MMP overexpression, preventing the occurrence of remodeling.^[^ ^39^ ^]^

### 3.3 Persistent Infection

Complex pathogenesis leads to the occurrence of persistent infections such as vasculopathy and immunopathy,^[^ ^5^, ^43^ ^]^ which adds to the complexity of DFU.

Vasculopathy causes hypoxia in tissues and limits immune cells from reaching the infected site through circulation. Immunopathy includes impairment of both intrinsic and adaptive immunity. In a hyperglycemic environment, wounds are more susceptible to infection;^[^ ^44^ ^]^ in addition, hyperglycemia affects the morphology and function of immune cells with weakened chemotaxis, phagocytosis, and microbial killing capability.^[^ ^43b^ ^]^ As a consequence, microbial flora (mainly bacteria) from the external environment as well as from the organism itself colonize the wound under the induction of various factors.^[^ ^45^ ^]^ And worse, the accumulation and adherence of multiple microorganisms stimulate the formation of biofilms, which are more difficult to treat than microorganisms.^[^ ^45^, ^46^ ^]^

The adverse effects of infections on healing are manifold. Continuous infection stimulates the secretion of inflammatory factors (e.g., TNF‐*α*) and proteases, which in turn prolong the presence of immune cells in the wound bed, degrade essential proteins (e.g., growth factors), and damage ECM remodeling. In addition, long‐term infection is toxic to critical for skin healing, manifested by unfavorable cell migration, disruption of mitochondrial function, and eventually induction of apoptosis. It also alters the wound pH and lactate deposition, thereby accelerating the formation of unfriendly microenvironments for DFU's healing.^[^ ^47^ ^]^

### 3.4 Hypoxia and Ischemia

As is well known that oxygen plays a key role in wound healing.^[^ ^48^ ^]^ After an injury, the wound is exposed to hypoxia, which promotes the initiation of wound healing processes, such as inflammatory response and angiogenesis.^[^ ^48a^ ^]^ Unlike transient hypoxia in acute wounds, DFU end up in a chronic hypoxic environment with oxygen tension usually under 20 mmHg, which is related to the imbalance between restricted oxygen supply (impaired vascular system) and high oxygen demand (required for cellular repair of wounds) in healing tissues.^[^ ^48^, ^49^ ^]^ Hypoxia‐inducible factor 1 (HIF‐1) is a pivotal regulator of oxygen homeostasis, affecting the expression of hundreds of genes. Under normal hypoxic conditions, HIF‐1 is activated, which stimulates the expression of downstream genes to promote erythropoiesis and produce growth factors (such as VEGF, PDGF, and basic fibroblast growth factor [bFGF]), which are essential for the regulation of hypoxia.^[^ ^49^, ^50^ ^]^ Notably, VEGF is one of the most potent proangiogenesis factors and impaired HIF‐1/VEGF axis function may be a crucial reason for DFU vascular damage.^[^ ^51^ ^]^ Unfortunately, under the influence of hyperglycemia and oxidative stress, HIF‐1/VEGF expression is downregulated in chronic wounds including DFU,^[^ ^49^, ^52^ ^]^ making angiogenesis insufficient and causing ischemia.

Given the necessity of oxygen for healing events, prolonged hypoxia makes DFU healing events more challenging. Wound repair demands a large supply of energy, however, ischemia and hypoxia aggravate the crisis in energy supply.^[^ ^10^ ^]^ Worse still, hypoxia is exacerbated by the massive, highly oxygen‐depleted inflammatory cells in the wound bed and the high metabolism of regenerating tissue.^[^ ^53^ ^]^

### 3.5 High Protease Activity

The wound site is full of proteases, especially MMP,^[^ ^54^ ^]^ which act as “decomposers” in the wound and are involved in all stages of healing. The secretion, activation, and homeostasis of MMP have an active role in wound healing.^[^ ^37^, ^54^ ^]^ In response to signals such as ROS, proinflammatory cytokines, and ECM, numerous cells in the wound secrete inactive MMP zymogens, such as neutrophils, fibroblasts, and keratinocytes.^[^ ^7^, ^37^ ^]^ Subsequently, MMP zymogens are converted to active MMP by serine proteases and kallikrein. MMP homeostasis is mainly regulated specifically by tissue inhibitors of metalloproteinases (TIMPs).^[^ ^37^, ^55^ ^]^ However, studies have demonstrated that MMP expression is upregulated but TIMP expression is downregulated in DFU.^[^ ^37^, ^54^ ^]^ In addition to endogenous proteases, microorganisms colonizing the wound periphery also express exogenous proteases to facilitate their proliferation.^[^ ^11^ ^]^ Overall, high levels of endogenous and exogenous proteases degrade essential proteins, resulting in exacerbated tissue necrosis in the wound with a large amount of protein exudate, leading to failure of wound healing.^[^ ^11^, ^54^ ^]^

### 3.6 Wide Range of pH

The intact skin surface is naturally acidic, influenced by the secretion of organic acids with a pH between 4 and 6 by epithelial cells, and acts as a natural barrier. From the surface, the pH of the skin increases with depth until ≈7.4.^[^ ^12^, ^32^ ^]^ When the skin is damaged, neutral tissues within the organism are exposed to the environment.^[^ ^12a^ ^]^ Thus, for acute wounds, pH regains acidity along with wound closure.^[^ ^12b^ ^]^ However, chronic wounds are usually accompanied by a more alkaline microenvironment than acute wounds depending on pathophysiology.^[^ ^32^ ^]^ According to a review by Schneider et al., chronic wounds with high bacterial burdens usually exhibit pH above 7.3.^[^ ^12a^ ^]^ Similarly, Jones et al. concluded that pH in chronic wounds ranged from 7.15 to 8.9.^[^ ^12b^ ^]^ However, pH up to 9.0 has also been recorded clinically with pH down to 6.41.^[^ ^12^, ^56^ ^]^ Therefore, the pH milieu of DFU is generally widely ranged and is a dynamic process, which is associated with diverse factors, such as the colonization of different microorganisms, the time of onset, severity, and wound stage.^[^ ^12a^ ^]^

Undoubtedly, restoring the natural acidity of the skin is conducive to wound healing. Because a more alkaline pH encourages microbial colonizing, coupled with the highest MMP activity and impaired fibroblast activity, as well as reduced oxygen perfusion and angiogenesis.^[^ ^57^ ^]^ Therefore, it is possible to predict wound status by monitoring wound pH clinically.^[^ ^12a^ ^]^

## 4 Nanomedical Strategies for DFU Treatment

Clinically, glycemic control runs through the course of treatment for DFU patients, which is usually combined with reduced pressure and offloading measures to attenuate DFU deterioration.^[^ ^58^ ^]^ However, these treatments are far from a complete cure of DFU. The complex microenvironment of DFU depicted above was considered a major influence factor that brings great challenges in DFU healing. The DFU clinical treatment modalities for different DFU microenvironments were summarized in **Table** **2**, which generally include regulating blood sugar in diabetics, anti‐infection, or protease inhibition to realize etiologic treatment and give maximum relief to the patient. However, the healing rate of DFU is still less than 50% and the recurrence rate is high.^[^ ^14d^ ^]^ How to address such issues and get a better outcome for DFU? Fortunately, nanomedicines have shown the potential for mitigating such shortcomings (Table 2). The results achieved so far in promoting wound healing by directly using nanomaterials or implanting them into wound dressings are quite encouraging. Aiming to advance the field of DFU nanomedicine, this section will discuss specific nanostrategies for modulating the microenvironment and the existence of limitations.

**Table 2:** Current treatment modalities and deficiencies in modulating the DFU microenvironment, and the potential of nanomedicines in mitigating DFU treatment shortcomings

Microenvironment	The main clinical treatment modalities	Current clinical treatment shortcomings	Mitigating shortcomings through nanomedicine	Examples of relevant nanomedicines
Hyperglycemia	Hypoglycemic drugs: such as insulin, metformin hydrochloride, etc.	Low bioavailability	Improving the physicochemical properties of drugs and controlling their release	Integrating copper‐based metal–organic frameworks (MOF) into thermosensitive hydrogels to enhance the bioavailability of curcumin and metformin hydrochloride^[^ ^59^ ^]^
Hyperinflammation	(1) Anti‐oxidative stress: such as alpha‐lipoic acid and curcumin; (2) gas therapy; (3) angiogenic drugs^[^ ^60^ ^]^	Nonspecific targeting, low bioavailability, negative effects of long‐term use of anti‐inflammatory drugs	(1) Microenvironment‐based targeted drug release; (2) development of immunomodulatory nanomedicines, such as ROS scavenging and modulation of immune cells	Curcumin nanoparticles with anti‐inflammatory and antioxidant properties accelerate wound healing^[^ ^61^ ^]^
Persistent Infection	(1) Antibiotics; (2) ddebridement to remove biofilm	(1) Drug‐resistant microbes and biofilm formation, reducing antibiotic efficacy; (2) debridement is painful and expensive for patients	Designing antibiotic‐free antimicrobial nanosystems	Metallo‐nanodrugs release zinc ions for antibacterial^[^ ^62^ ^]^
Hypoxia and ischemia	(1) Hyperbaric oxygen therapy and topical gaseous oxygen; (2) proangiogenic drugs, such as growth factors; (3) severe ischemia requires hemodynamic reconstruction	(1) Inability to sustain oxygen levels; (2) high cost; (3) bioactive ingredients are susceptible to degradation and inactivation	Incorporating therapeutic agents into carriers to prevent protease degradation and control release	A multifunctional hydrogel that acts as a protective barrier delivering manganese dioxide (MnO_2_) nanosheets, exosomes, and FGF to mitigate hypoxia and promote tissue repair^[^ ^63^ ^]^
High protease	(1) Traditional dressing to absorb leachate; (2) inhibitors of metalloproteinases, e.g., tetracyclines and collagen dressings^[^ ^64^ ^]^	(1) Single function of traditional dressing; (2) tetracycline resistance; human‐derived collagen is expensive and denatured collagen is easily degraded^[^ ^64b^ ^]^	(1) Designing multifunctional wound dressings to encourage wound healing; (2) delivering genes to silence MMP expression	Chitosan‐based multifunctional nanofibre dressing with antibacterial, antioxidant, and wound leachate absorption capabilities^[^ ^65^ ^]^
Wide range of pH	Honey (pH = 3.4–4.5): antibacterial and prevents degradation of ECM by proteases^[^ ^66^ ^]^	(1) The complex composition of honey makes the relationship between pH and efficacy difficult to determine; (2) insufficient evidence on whether honey is good for wound healing	Development of pH‐responsive based nanomedicines to modulate wound pH	pH‐switchable iron oxide nanoenzymes exert catalase and peroxide activity in neutral wound tissue and acidic biofilms, respectively, to alleviate hypoxia and biofilm infection^[^ ^67^ ^]^

### 4.1 Downregulation of Blood Glucose

Unsatisfactory glycemic control is the main culprit of DFU.^[^ ^58^ ^]^ Normal venous blood glucose value is less than 6.1 mmol L^−1^ or less than 7.7 mmol L^−1^ within 2 h after a meal. However, in diabetic patients, random blood glucose values are usually greater than 11.1 mmol L^−1^ (great individual differences).^[^ ^38^, ^68^ ^]^ Downregulation of blood glucose is beneficial in mitigating the progression of microvascular disease and diminishing vascular complications (including neuropathy) in patients to alleviate pain and improve quality of life.^[^ ^69^ ^]^ Clinically, insulin, metformin, dipeptidyl peptidase‐4 inhibitors, and *α*‐glucosidase inhibitors are good hypoglycemic candidates for DFU patients. Nevertheless, the low bioavailability of most drugs often results in a poor therapeutic effect. Based on the capability of nanomaterials to modulate drug release to enhance bioavailability, payloading hypoglycemic drugs in nanocarriers is an effective method to remodel the DFU hyperglycemic microenvironment into normoglycemia. Therefore, Yang et al. constructed copper‐based MOF nanoparticles (HKUST‐1 NPs) loaded with metformin hydrochloride and the anti‐inflammatory agent curcumin (Cur/MH/HKUST‐1 NPs), and subsequently infiltrated Cur/MH/HKUST‐1 NPs into the hydrogel (Cur/MH/HKUST‐1@Gel) to reformat the microenvironment in DFU. Interestingly, blood glucose values (greater than 16.7 mmol L^−1^) in diabetic mice descended 7.2 mmol L^−1^ after 20 d of Cur/MH/HKUST‐1@Gel treatment, which was similar to the metformin treatment (8 mmol L^−1^).^[^ ^59^ ^]^ Except for metformin hydrochloride, insulin was also loaded into nanosystems to promote wound healing. Accordingly, insulin‐loaded micelles were established and further embedded in the hydrogel with EGF. The drug loading content of insulin in micelles was 25.6%, and the insulin released at the wound effectively reduced the blood glucose level within 6 d.^[^ ^70^ ^]^ Notably, despite satisfactory results in mouse models, highly hydrophilic therapeutics payloading remains challenging for researchers to construct a suitable nanodelivery system to meet the clinical therapeutic requirement.

**Figure 3:**
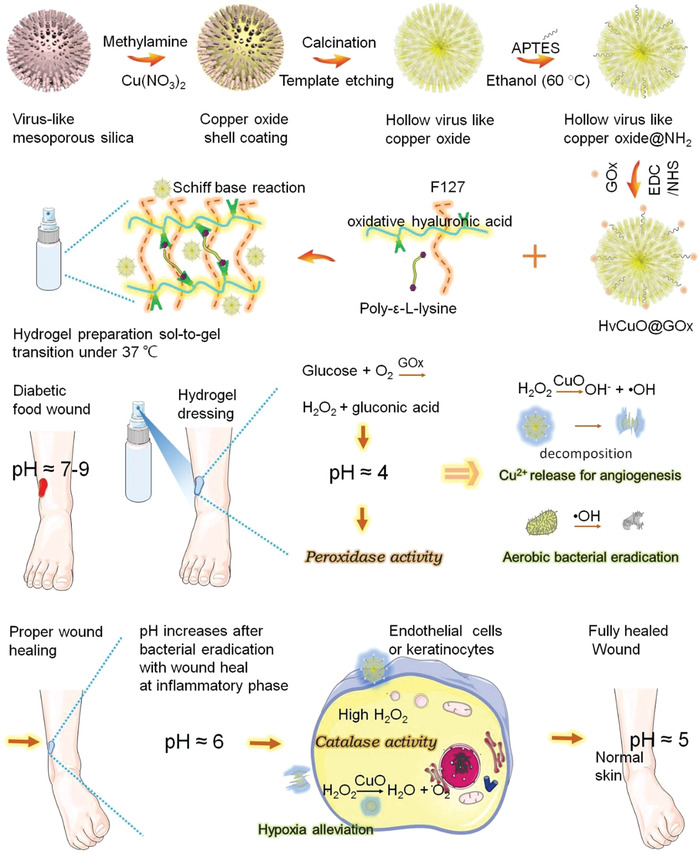
Using a glucose‐initiated cascade reaction, GOX modified virus‐like hollow mesoporous CuO nanoparticle based hydrogel spray (HvCuO@GOX) could remodel the microenvironment of hyperglycemia, pH abnormalities, deficient angiogenesis, infection, and hypoxia, and further achieve wound closure. Reproduced with permission.^[^ ^71^ ^]^ Copyright 2021, Elsevier.

Alternatively, nanosystems with glucose oxidase (GOX)‐like activity can also achieve hypoglycemic effects through decomposing glucose. Typically, a recent study fabricated hydrogels of GOX hybrid virus‐like hollow mesoporous copper oxide (HvCuO@GOX) NPs. In which, glucose was firstly decomposed by GOX to produce gluconic acid and hydrogen peroxide (H_2_O_2_). Then, the generated gluconic acid reduced the pH of the wound to ≈4.0, further inducing the peroxidase‐like activity of CuO (catalyzing H_2_O_2_ to hydroxyl radicals [•OH]) to exert antibacterial activity. The bacterial eradication subsequently elevated the pH to ≈6.0, which stimulated the catalase activity of CuO (catalyzing H_2_O_2_ to oxygen) to relieve hypoxia in the wound bed (**Figure** **3**).^[^ ^71^ ^]^ Similarly, Li et al. used a MOF to load GOX and bovine hemoglobin (BHb) with peroxidase‐like activity and achieved hypoglycemic and antibacterial effects.^[^ ^72^ ^]^ However, it should be noticed that the underlying toxicity of metal ions and organic ligands from MOF materials should be examined for general applications.

More, Bhadauriya et al. produced carbon nanofibers containing yeast extract and Cu NPs. The yeast extract enhanced glucose consumption by converting glucose to ethanol. Thus, it was observed that the glucose concentration decreased from 8.33 to 6.94 mmol L^−1^ in less than 12 h after using the nanofiber. In addition, Cu NPs imparted antibacterial activity to this system based on various mechanisms (permeating bacterial cell membranes, damaging DNA, etc.). More encouragingly, Cu NPs also facilitated angiogenesis, fibroblast proliferation, and collagen formation, which was associated with the properties of stabilizing the HIF‐1*α* expression and VEGF secretion.^[^ ^73^ ^]^ Overall, Cu, as the indispensable trace element in organisms, features various biomedical effects due to its intrinsic physicochemical properties. Therefore, it is needed to construct Cu‐based nanosystems rationally to overcome the potential ionic toxicity and satisfy the clinical requirements.

Beyond the hypoglycemic strategies described above, interestingly, we can also focus on nanostrategies for diabetic treatment to expand our arsenal of DFU nanomedicines. For example, “carbon monoxide nanogenerators” featuring anti‐inflammatory and anti‐apoptotic properties decrease blood glucose by protecting B cells from damage.^[^ ^74^ ^]^ Besides, microneedles are utilized to regulate blood glucose in diabetic mice through transdermal delivery of insulin‐loaded nanoparticles, coupled with GOX and catalase co‐loaded nanoparticles.^[^ ^75^ ^]^ In short, nanomedicines for diabetes care have been extensively investigated, which will pave a new pathway for the development of DFU nanomedicines.

### 4.2 Promotion of Hemostasis

Hemostasis is the first step in wound healing, and some wounds have a small amount of blood loss. Therefore, materials with hemostatic properties are needed for wound closure.^[^ ^76^ ^]^ According to reports, the material can accomplish hemostasis by absorbing blood, activating platelets, encouraging blood coagulation, etc. Correspondingly, various active ingredients have exhibited excellent hemostatic capabilities, such as chitosan, gelatin, fibrin, hyaluronic acid (HA), silicon dioxide (SiO_2_), and zinc oxide (ZnO).^[^ ^20^, ^77^ ^]^ Among them, fibrin, as a natural coagulation protein, promotes blood clotting and activates platelets to exert hemostatic functions. Therefore, Mohandas et al. formulated a composite sponge based on VEGF‐loaded fibrin NPs and demonstrated that this sponge had a superior capacity to stimulate blood clotting from the ulnar vein than the commercially Calcium Alginate Sodium Dressing, Kaltostat through blood clotting studies.^[^ ^76^ ^]^ In another study, hydrazide‐grafted HA (HAh) was constructed into injectable hydrogels by crosslinking with aldehyde and quaternary ammonium‐grafted HA (HAaq) through Schiff base formation. The rapid gelation of the hydrogel at the wound endowed it with the hemostatic ability and, in addition, its excellent hemostatic function also derived from attracting blood cells electrostatically through the positively charged quaternary ammonium group of the hydrogel.^[^ ^63^ ^]^ However, the cytotoxicity of the remaining aldehyde groups is a prevalent obstacle with Schiff base‐induced adhesive hydrogels. Therefore, careful computation of the —NH_2_/—CHO proportion is desired to equilibrate cell viability and adhesive function.

### 4.3 Anti‐inflammatory

*α*‐lipoic acid and acetyl‐l‐carnitine are considered as potential antioxidants in the management of diabetic peripheral neuropathy (DPN) clinically. However, their antioxidant capacity does not appear to completely improve DPN prognosis.^[^ ^78^ ^]^ Therefore, the next stage requires exploring effective therapies. Recent studies have uncovered the potential application of nanosystems for anti‐inflammatory therapy. Accordingly, the nanosystem should meet at least one of the following conditions to achieve anti‐inflammation. And, the corresponding treatment strategies are detailed in **Table** **3**.

- promoting the macrophages polarization to M2;
- down/upregulating pro/anti‐inflammatory cytokines;
- downregulating ROS.

**Table 3:** Strategies of nanosystems to regulate inflammation

Strategies		Examples	Mechanisms
Promoting the macrophages polarization to M2	Modulating M2‐related marker	Konjac glucomannan‐modified silica NPs^[^ ^79^ ^]^	Clustering mannose receptors on the surface of macrophages
	Delivering nitric oxide (NO)	NO‐loaded HKUST‐1 particles^[^ ^83^ ^]^	An appropriate concentration of NO processes the ability of polarizing macrophages from M1 type to M2 type (via NO/VASP pathway)
	Delivering cytokines	IL@ZIF nanoplatform^[^ ^84^ ^]^	IL‐33 upregulates the level of its receptor ST2L, and then, utilizing the IL‐33/ST2L signal stimulates the polarization of macrophages
	Delivering stem cells	Biomaterials containing adipose‐derived stem cells (ADSCs)^[^ ^16b^ ^]^	ADSCs can: secrete various chemical signals (such as growth factors and chemokines) to reverse the wound pathological microenvironment; differentiate into cells with repair capability; and contribute to the overall process of wound healing^[^ ^85^ ^]^
	Utilizing exosomes	HA@MnO_2_/FGF‐2/Exos^[^ ^63^ ^]^	Exosome‐carried “cargo” (miRNA‐233) induces phenotypic transformation of macrophages
	Delivering ATP	ATP‐vesicles,^[^ ^86^ ^]^ nanoliposome‐encapsulated Mg‐ATP^[^ ^87^ ^]^	ATP promotes cell recruitment, adenosine mediates macrophage phenotypic transformation, and so on^[^ ^10^ ^]^
	Utilizing high‐molecular‐weight hyaluronic acid	M2 macrophage‐polarized anti‐inflammatory hydrogel^[^ ^88^ ^]^	HA stimulates M2 phenotype‐specific gene expression
Regulating pro/anti‐inflammatory cytokines	Delivering stem cells	Hydrogel‐encapsulated ADSCs^[^ ^89^ ^]^	Consistent with the above stem cells that promote macrophage polarization
	Gene therapy	MiRNA‐146a‐conjugated cerium dioxide (CeO_2_) NPs^[^ ^90^ ^]^	The expression of miRNA‐146a was downregulated in DFU, resulting in increased expression of pro‐inflammatory cytokines IL‐6 and IL‐8/MIP‐2. Therefore, exogenous delivery of miRNA‐146a decreases the level of related factors
		Lipidoid NPs loaded with siRNA (silencing TNF‐a)^[^ ^91^ ^]^	Reducing TNF‐a gene transcription and translation levels, thereby regulating the inflammatory microenvironment
	Delivering Antioxidants	Chitosan‐loaded neurotensin^[^ ^92^ ^]^	Neurotensin is an inflammatory regulator that reduces TNF‐*α* levels in the wound, which in turn reduces MMP‐9 levels, prompting wound repair to enter a proliferative phase
	Self‐activity of materials	Chitosan‐coated CeO_2_ nanocubes^[^ ^93^ ^]^	CeO_2_ NPs can promote the expression of the anti‐inflammatory factor IL‐10 and inhibit the proinflammatory factor TNF‐*α*
		Heparin‐based hydrogel^[^ ^94^ ^]^	Absorbing pro‐inflammatory chemokines
Downregulating ROS	Self‐activity of materials	Niacin MOFs^[^ ^95^ ^]^	Zn^2+^/Cu^2+^ activates the endogenous antioxidant enzyme (superoxide dismutase)
		CeO_2_^[^ ^96^ ^]^	CeO_2_ NPs emulate superoxide dismutase to scavenge free radicals
		MnO_2_ nanoenzymes^[^ ^63^ ^]^	Simulation of catalase
		Ultrasmall Cu_5.4_O nanoparticles^[^ ^94^, ^97^ ^]^	Mimicking multiple antioxidant enzymes
		tempol‐grafted, manganese‐doped, mesoporous silica nanoparticles (TMSN)^[^ ^98^ ^]^	The synergy between the radical‐scavenging capacity of tempol and H_2_O_2_ breakdown ability of MSN
	Carrying anti‐inflammatory drugs or antioxidants	Curcumin@ZIF‐8 MOFs^[^ ^99^ ^]^	Curcumin can scavenge free radicals and also activate the endogenous anti‐inflammatory Nrf2 pathway
		Gelatin‐Tannic acid‐Alla hydrogel^[^ ^100^ ^]^	Both tannic acid and Alla are natural antioxidants, with the ability to scavenge free radicals. Of them, tannic acid has a stronger antioxidant capacity owing to its polyphenol structure
		Hydrogels loaded with superoxide dismutase^[^ ^101^ ^]^	Scavenging ROS by releasing superoxide dismutase in the wound
	Gene therapy	Novel lipoproteoplex‐loaded Keap1 siRNA^[^ ^102^ ^]^	Degradation of the anti‐inflammatory factor Nrf2 can be inhibited by silencing Keap1 expression, which is atypically upregulated in DFU. Silencing of Keap1 can reverse ROS imbalance through Nrf2‐mediated endogenous antioxidant pathway
	Delivering hydrogen gas	Photodriven nanoreactor for delivery of gaseous hydrogen and insulin^[^ ^18r^ ^]^	Hydrogen triggers the Nrf2 signaling pathway, which helps to scavenge excess ROS. Meanwhile, hydrogen reactivates insulin signaling, thereby enhancing antioxidant benefits

**Figure 4:**
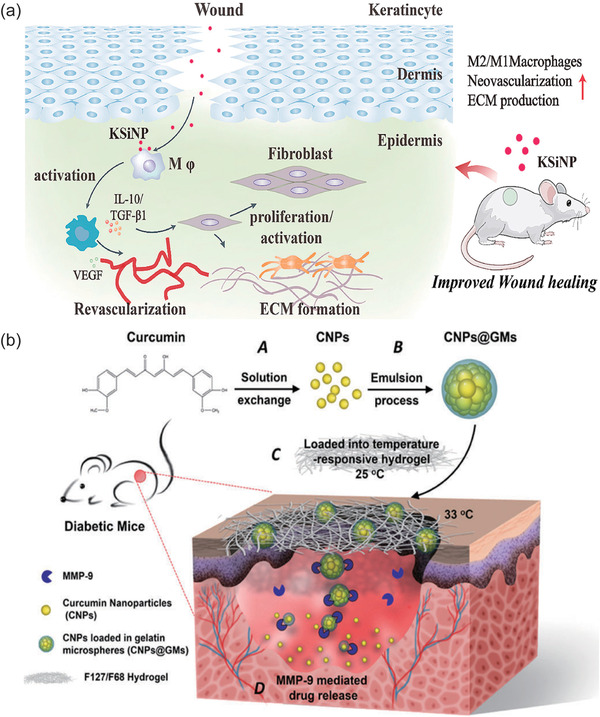
a) Konjac glucomannan‐modified SiO_2_ nanoparticles (KSiNPs) promote M2‐type polarization of macrophages by aggregating mannose receptors on cells. Reproduced with permission.^[^ ^79^ ^]^ Copyright 2019, Elsevier. b) Schematic representations of CNPs@GMs/hydrogel preparation and the process of drug release at the wound bed in diabetic mice. A) Preparation of pure CNPs via a solution exchange method. B) CNPs loaded into GMs by the emulsion process to get CNPs@GMs. C) CNPs@GMs mixed with the thermos‐sensitive hydrogel and covered on the wound in diabetic mice. D) Under the microenvironment of a nonhealing wound, GMs were degraded by MMPs, and specifically the drug was specifically released. Reproduced with permission.^[^ ^61^ ^]^ Copyright 2018, Elsevier.

To satisfy *Condition I*, nanosystems should possess the competence to downregulate M1 type macrophage markers (iNOS2, CD86, etc.) or upregulate M2 type markers (CD206 (mannose receptor), CD163, arginase, etc.). Further strategic studies have illustrated that nanosystems facilitate macrophage polarization by delivering NO, stem cells, cytokines, etc. For example, konjac glucomannan‐modified silica NPs with a size of 30 nm could trigger mannose receptor clustering on the surface of macrophages and promote M1 polarization to M2 (**Figure** **4**a).^[^ ^79^ ^]^ This “cargo‐free” material itself has the activity to close wounds, inspiring us to design materials with simple components and high activity for biomedical applications.

To satisfy *Condition II*, multifunctional nanosystems have been designed to load antioxidants, genes, or others. For instance, natural anti‐inflammatory molecules hypaphorine were encapsulated into W/O‐type NPs with chitosan and demonstrated to downregulate the levels of IL‐1*β* and TNF‐*α* in the wound model of diabetic rats, further accelerating the tissue regeneration.^[^ ^80^ ^]^

To satisfy *Condition III*, the nanosystem requires the capability to neutralize ROS and/or increase antioxidant enzyme levels to meet therapeutic needs. Given the critical regulatory role of ROS in physiological function, considerable effort has been devoted to ROS scavenging. ROS‐based nanomedicine has become a hot research topic and is continuously explored for better guiding clinical applications. Correspondingly, a nanocomposite hydrogel was created based on anionic biopolymer alginate and positively charged eudragit nanoparticles incorporating edaravone. On one hand, the edaravone possesses a powerful •OH scavenging function and has clinical application in the treatment of acute cerebral infarction. On the other hand, the eudragit nanoparticles could surmount bottlenecks in the administration of edaravone via increasing its stability and solubility. Thus, this nanocomposite hydrogel could promote wound healing in diabetic mice by regulating the ROS level in a dose‐dependent manner.^[^ ^81^ ^]^ In addition to edaravone, other ROS scavengers, such as curcumin, possess anti‐inflammatory abilities to reduce ROS and elevate antioxidant enzyme (glutathione peroxidase) levels. As displayed in Figure 4b, curcumin NPs were encapsulated into gelatin microspheres (CNPs@GMs) in response to MMP at the DFU wound. Then, CNPs@GMs were further enveloped into a thermo‐sensitive hydrogel for sustained release of curcumin to promote wound healing.^[^ ^61^ ^]^

Since various signals in the organism are complex and interactive, the above three conditions might affect each other to a certain extent. Nevertheless, we believe that these conditions underlie anti‐inflammatory strategies. Noticeably, certain inevitable limitations make the clinical transition of such nanosystems from bench to bedside difficult. One overriding challenge is most likely the redox pathology of DFU. Even in the same inflammatory disease, the state of redox changes over time and the degree of onset, which bothers the frequency, dosage, and therapeutic effect of nanomaterials. The ideal antioxidant therapy should balance redox homeostasis well, rather than excessive or inadequate inhibition of oxidative stress.^[^ ^82^ ^]^ To this end, scientists ought to develop simpler, faster, and more sensitive systems for quantifying oxidative stress in vivo to overcome this limitation.

### 4.4 Anti‐infection

Antibiotics are the most potent agents known to combat infections clinically. However, questions have been rising about the emergence of drug‐resistant microorganisms. Encouragingly, advanced nanotechnology has facilitated innovation in therapeutic strategies, enabling the preparation of various versatile nanomaterials for anti‐infection therapy, such as silver (Ag), gold (Au), Cu, ZnO, graphene oxide (GO), and GO‐Ag nanocomposite.^[^ ^103^ ^]^ As mentioned previously, wounds in diabetic patients are more susceptible to infection, especially from bacteria. Generally, the most common gram‐positive bacteria isolated in DFU are *Staphylococcus aureus* (*SA*) and gram‐negative bacteria are *Escherichia coli* (*EC*) or *Pseudomonas aeruginosa* (*PA*). Therefore, antibacterial experiments in vitro and in vivo can be applied to evaluate the antimicrobial properties of nanomaterials, and they can treat infections via their inherent antimicrobial properties or acting as vehicles for antimicrobial agents.

One of the most typical examples is Ag NPs, which can adhere to or penetrate the cell wall/membrane, inhibit bacterial cell respiration, and destroy protein structures.^[^ ^80^, ^104^ ^]^ Recent evidence has also demonstrated that Ag stimulates the function of the immune system, which may be associated with the activation of the inflammasome, resulting in the production of large numbers of neutrophils and white blood cells to promote bacterial clearance.^[^ ^105^ ^]^ More encouraging, Krishnan et al. further evidenced that Ag NPs could reduce the level of MMP‐2 and MMP‐9 at the wound.^[^ ^106^ ^]^ Such function may be related to the down‐regulation of TNF‐*α* with bacterial elimination, although showing concentration dependence (maximum 25 ppm).^[^ ^107^ ^]^ Overall, the versatile Ag NPs require us to continuously explore their pharmacological toxicology for optimal clinical governance.

Meanwhile, bacterial infections tend to exacerbate inflammation. To this end, a great number of nanomaterials have been applied to target bacteria to sterilize and reduce the inflammation they cause.^[^ ^108^ ^]^ For instance, Peng and co‐workers formulated platelet‐membranes‐coated mesoporous copper silicate microspheres (CSO@PM). Utilizing the properties of platelet membranes to target bacteria and adsorb endotoxins along with the photothermal properties of CSO, CSO@PM serves as a multifunctional antimicrobial platform for the antimicrobial and mitigation of inflammation induced by lipopolysaccharides from gram‐negative bacteria.^[^ ^108a^ ^]^

**Figure 5:**
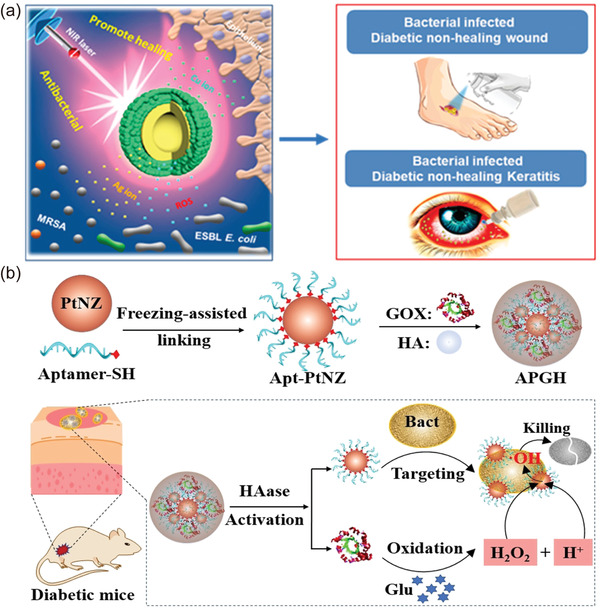
a) Near‐infrared (NIR)‐activated AuAgCu_2_O nanoshells for antibacterial‐resistant bacterial killing and improved wound healing. Reproduced with permission.^[^ ^109a^ ^]^ Copyright 2020, American Chemical Society. b) GOX catalyzes glucose to lower the pH of the wound and provide H_2_O_2_, which activates the nanoenzyme to produce ROS for sterilization. Reproduced with permission.^[^ ^111^ ^]^ Copyright 2021, Wiley‐VCH.

In addition, the nanosystem generates local high temperatures to induce photothermal ablation of bacteria by utilizing the photothermal effect of nanomaterials themselves or carrying a photothermal agent under laser irradiation.^[^ ^109^ ^]^ Correspondingly, Qiao et al. designed a cupriferous hollow nanoshell constructed from a hollow Au–Ag core and CuO shell. Such a nanoshell possessed a photothermal effect and the ability to eradicate multi‐resistant bacteria, which favored the pro‐epithelialization and promoted DFU healing effectively (**Figure** **5**a).^[^ ^109a^ ^]^

Nanosystems for ROS production have also been explored to attack bacteria, given the destruction of cell membranes, proteins, and even DNA by ROS. A prominent example is that nanomaterials with peroxidase‐like activity have been developed to generate ROS through catalytic reactions. Li et al. designed a GOX‐loaded ionic covalent organic framework (iCOF) with peroxidase activity. The GOX promoted glucose consumption and produced H_2_O_2_, which was further catalyzed to •OH in the iCOF to kill *EC* and *SA*.^[^ ^110^ ^]^ Similarly, Chen et al. prepared a nanozyme capsule (APGH) with HA as a shell for loading aptamer‐functionalized platinum nanozymes (Apt‐PtNZ) and GOX. Once the HA shell was degraded by hyaluronidase generated by bacteria, the releasing Apt‐PtNZ targeted bacteria (*SA*) through aptamer binding and exerted peroxidase activity. Thus, the APGH exerted hypoglycemic, pH‐lowering, and sterilizing effects (Figure 5b).^[^ ^111^ ^]^ Besides, photodynamic therapies (PDT) that rely on photosensitizers and light sources can also generate ROS for infection treatment. Correspondingly, the photosensitizer prussian blue based nanosystem was designed to effectively eradicate biofilms in vitro.^[^ ^112^ ^]^

Being the carriers of antimicrobial agents is another important application of the antimicrobial effect of nanomaterials, such as antibiotics, antimicrobial peptides, NO, and other “cargoes” with bactericidal effects.^[^ ^96^, ^113^ ^]^ Specifically, the antimicrobial peptide LL37 was implanted into Au NPs containing VEGF plasmids (AuNPs@LL37/pDNAs) for infection and angiogenesis therapy. Antibacterial assays illustrated that AuNPs@LL37/pDNAs had comparable antibacterial effects to vancomycin and could kill almost all *MRSA* bacteria. Furthermore, combined with the proangiogenic ability of VEGF, the nanosystem displayed good therapeutic potential in ulcer treatment.^[^ ^113a^ ^]^

Of note, the presence of bacteria and biofilm infection in the wound bed can induce an immune response in the host, however, the microbicidal activity of immune cells (e.g., neutrophils and macrophages) is often suppressed due to complex biofilm‐host mutual immunoregulation.^[^ ^114^ ^]^ Consequently, some multifunctional patches composed of nanomaterials that can potentiate the immune system appear to be emerging as attractive antibacterial tools.^[^ ^62^, ^115^ ^]^ A representative example is a composite patch implanted with superparamagnetic iron oxide nanoparticles (SPIONs) that can induce M2 to M1 type polarization of macrophages, thereby removing bacteria and biofilms. Besides, this patch, prepared from collagen, hyaluronic acid, chitosan, etc., presented an environment conducive to wound healing, while introducing FSTL‐1 protein/AC2‐26 peptide to facilitate angiogenesis and epithelialization/reduce inflammation, respectively.^[^ ^115^ ^]^ Presumably, such versatile patches are highly potential tools for clinical translation.

However, despite much work highlighting the role of nanomaterials in antimicrobial applications, it is undeniable that silver NPs are the only nanomaterials commercially available in antimicrobial dressing products. The toxicity of nanomaterials remains a paramount challenge in moving towards clinical implementation, and the particle size, shape, charge, and composition of nanomaterials are all known to be related to toxicity. Additionally, for a better evaluation of efficacy, more effective small or large animal models should be designed. Namely, persistent chronic bacterial infections and biofilm infections do exist in relevant models. In parallel, overcoming antibiotic resistance is a predominant justification for the exploitation of non‐antibiotic medicines.^[^ ^116^ ^]^ Thus, in‐depth research into the underlying development of resistance to these nanomedicines is highly desirable for further applications.

### 4.5 Regulate Angiogenesis

Guided angiogenesis can effectively reformulate the microenvironment of ischemia in DFU. The formation of blood vessels depends on the interaction of a series of growth factors and cells, among which the most important are VEGF and endothelial cells. Growth factors combine with specific receptors on the cell surface to regulate cellular function by activating relevant signals, and the same growth factor can induce distinct cellular responses depending on the microenvironment and cell type. Chronic wounds often exhibit low expression of growth factors, yet they are essential for angiogenesis, regulation of inflammation, and promotion of EC remodeling and re‐epithelialization.^[^ ^117^ ^]^ Besides, angiogenesis is also regulated by other signals, such as oxygen, NO, and hydrogen sulfide (H_2_S).^[^ ^48^, ^118^ ^]^ Accordingly, many clinical studies have been conducted on the topical growth factor therapy for DFU such as PDGF, VEGF, and bFGF, although PDGF is the only one approved by FDA. Plus, gas therapy or other new therapies have been extensively explored.^[^ ^64a^ ^]^ Given the intelligent nature of nanosystems, some functional nanomaterials present significant synergistic benefits to deliver such therapeutics to the lesion sustainably. Also, some nanomaterials themselves possess therapeutic functions. To assess the proangiogenic ability of nanomaterials, the migration ability of endothelial cells is often determined using scratch tests in vitro, and the expression level of CD31 (a marker of angiogenesis) is often quantified in vivo. In general, nanosystems can regulate angiogenesis at the wound in the following manners.

**Figure 6:**
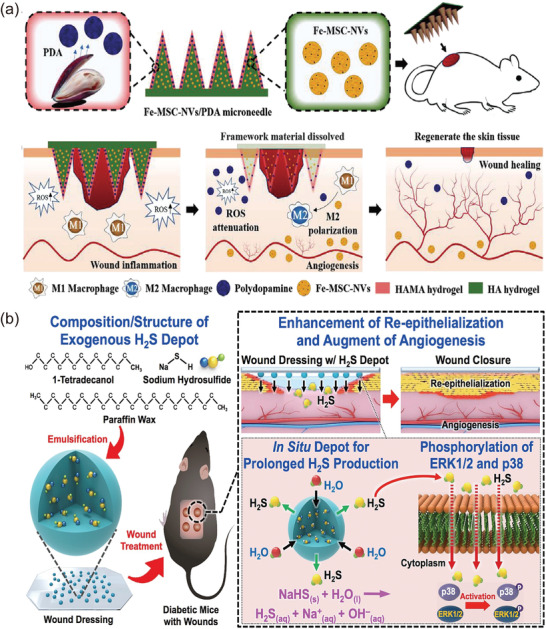
a) Treatment protocol of Fe‐MSC‐NV/PDA microneedle for DFU. Reproduced with permission.^[^ ^125c]^ Copyright 2022, Wiley‐VCH. b) The process of using NaHS@MPs to provide H_2_S to wounds and the mechanism of H_2_S promoting wound healing. Reproduced with permission.^[^ ^118c^ ^]^ Copyright 2017, Elsevier.

First, growth factors are delivered for direct upregulation, or indirect upregulation by delivering their “stimulants.” In the direct scenario, substrates such as nanofibers,^[^ ^119^ ^]^ and hydrogels^[^ ^120^ ^]^ were chosen for the delivery of cytokines such as VEGF^[^ ^121^ ^]^ and PDGF.^[^ ^119^, ^122^ ^]^ In the indirect scenario, the expression of the relevant factors is upregulated by the “cargoes” delivered or the activity of nanomaterials themselves. These “cargoes” contain therapeutic peptides (upregulating angiogenic genes and proteins in endothelial cells),^[^ ^123^ ^]^ desferrioxamine and dimethyloxallyl glycine (upregulating HIF‐1 levels),^[^ ^52^, ^124^ ^]^ exosomes (providing a wide range of growth factors and micro‐RNAs),^[^ ^125^ ^]^ engineered bacteria (engineering high VEGF expression),^[^ ^126^ ^]^ stem cells (recruiting growth factors),^[^ ^16b^ ^]^ and genes (increasing the expression of angiogenesis‐related cytokines).^[^ ^127^ ^]^ In a typical study, ferrum‐mesenchymal stem cell‐derived artificial exosomes (Fe‐MSC‐NVs) were implanted in microneedles with polydopamine (PDA) NPs to overcome the skin barrier. The Fe‐MSC‐NVs carried numerous cytokines, such as VEGF, HIF‐1*α*, and FGF2. These factors contributed to the proliferation and migration of endothelial cells, and thus, the expression of CD31 was notably upregulated in the diabetic mouse model. Furthermore, combined with the antioxidant capacity of the PDA, this nanosystem effectively reshaped the microenvironment of inflammation and insufficient angiogenesis in DFU (**Figure** **6**a).^[^ ^125c^ ^]^ In addition, among the schemes that exploit the activity of nanomaterials themselves, Cu‐containing NPs have been widely utilized to promote angiogenesis.^[^ ^109a^ ^]^ In addition, Mg‐containing NPs,^[^ ^128^ ^]^ bioactive glass NPs,^[^ ^129^ ^]^ fayalite,^[^ ^130^ ^]^ black phosphorus (BP) nanosheets,^[^ ^109c^ ^]^ and bioactive glasses^[^ ^131^ ^]^ have also been used to regulate angiogenesis.

Second, nanosystems promote angiogenesis by upregulating NO levels at the wound. NO in the organism is derived from arginine, which is catalyzed by nitric oxide synthase (NOS) to produce NO.^[^ ^132^ ^]^ NO is a significant signaling molecule in the body and can impact the functions of biological systems.^[^ ^83^ ^]^ For DFU wounds, low levels of endothelial NOS and NO expression are part of the pathology of vascular lesions.^[^ ^133^ ^]^ Therefore, NO‐releasing nanosystems have been extensively studied. For example, NO‐loaded Cu‐MOF materials could synergy with Cu ions from MOF to enhance vascular function.^[^ ^83^ ^]^ Furthermore, arginine‐loaded CeO_2_ NPs improved low levels of NO expression at the wound by utilizing arginine metabolism.^[^ ^134^ ^]^ Nonetheless, despite NO acting as a “star” messenger of physiological activity, a critical issue is the inability to accurately control concentration in clinical application. Low concentrations of NO (1 × 10^−6^ to 1 × 10^−3^ m) are therapeutically beneficial, whereas high concentrations (>1 × 10^−3^ m) might trigger underlying NO poisoning.^[^ ^135^ ^]^ Consequently, an in‐depth evaluation of NO release from the above or similar strategies is desired to develop a more beneficial platform for controllable release.

Third, H_2_S, an endogenous gas transmitter, has a proangiogenic function. H_2_S promotes the activation of endothelial cells and keratinocytes, which is related to the prolonged activation of ERK1/2 and p38 by H_2_S. Similar to NO, the effects of H_2_S are concentration‐dependent, i.e., high concentrations are potentially toxic. However, a clinical study revealed that plasma H_2_S levels were notably lower in diabetics, relative to the healthy group (45.1 vs 54.0 × 10^−6^ m).^[^ ^136^ ^]^ Hence, raising the level of H_2_S is a promising treatment for DFU. To surmount the delivery conundrum, NaHS, as an exogenous donor of H_2_S, was incorporated into microparticles (NaHS@MPs) and subsequently infiltrated NaHS@MPs into the film dressing. NaHS could take advantage of the moist environment to continuously convert to H_2_S with instantaneous release concentrations of 2–10 × 10^−6^ m in 12–48 h (Figure 6b).^[^ ^118c^ ^]^ Nevertheless, despite the significant biological function of H_2_S, especially in the skin,^[^ ^137^ ^]^ little attention has been devoted to the combination of nanotechnology with H_2_S in diabetic wound repair, which could be a potential research direction.

Notably, angiogenesis is frequently prevented by an excess inflammatory response, which exhibits a downregulation of NO levels with endothelial dysfunction.^[^ ^39^ ^]^ Thus, mitigating inflammation is crucial for promoting angiogenesis (see Section 4.3).^[^ ^60^, ^126^ ^]^ In addition, angiogenesis is tightly linked to the improvement of hypoxia (see Section 4.8).

Taken together, the advances in pro‐angiogenesis by nanomedicines are quite encouraging. However, several challenges are also worth noting. As reported, many nanomaterials exert proangiogenic effects at low doses, while higher doses are often antiangiogenic or even toxic (i.e., bimodal effects).^[^ ^138^ ^]^ In addition, the pro/antiangiogenic effect is also linked to the physicochemical properties of nanomaterials, such as size and charge. It should also be mentioned that therapeutic components (e.g., ions, gases) released from some nanomaterials may penetrate into nontarget tissues or organs, causing unnecessary and unpredictable biological side effects. Thus, the therapeutic effects of nanomaterials need to be carefully evaluated to well match needs.

### 4.6 Promotion ECM Remodeling

The production of ECM at the wound originates from fibroblast activity. The ECM is composed of several proteins, of which collagen (expressed by fibroblasts) is the most abundant and is closely related to ECM remodeling.^[^ ^30^, ^139^ ^]^ As mentioned before, proteases that are highly expressed in the DFU microenvironment degrade proteins, causing abnormal remodeling of the ECM and further impairing granulation tissue formation. Based on these, nanosystems can remodel the ECM through the following three strategies:

- promoting the migration and proliferation of fibroblasts;
- delivering collagen or its related substances exogenously;
- inhibiting the high expression of MMP in wounds.

For the *Condition I*, stem cells,^[^ ^16b^ ^]^ growth factors,^[^ ^121^ ^]^ and exosomes^[^ ^125a^ ^]^ can be delivered, or the power evoked by the nanomaterials themselves can be harnessed. These nanosystems facilitate the upregulation of growth factors to activate fibroblasts, which ultimately reduces side effects compared to direct administration. For example, nanomaterials loaded with VEGF and bFGF were implanted into a fibrin‐based scaffold to optimize growth factor therapy. This scaffold could significantly stimulate wound closure by slowly releasing VEGF and bFGF, which facilitated the activation of endothelial cells and fibroblasts/keratinocytes.^[^ ^121^ ^]^ Similarly, a nanofibrous scaffold synthesized for mimicking ECM and used for delivering VEGF and bFGF. At the animal level, substantial angiogenesis was observed, and, the expression levels of collagen I, collagen III, and Ki‐67 genes were upregulated in the tissues, demonstrating the therapeutic effectiveness of the scaffold.^[^ ^140^ ^]^

For the *Condition II*, recently, a pH and inflammation‐responsive hydrogel was designed as a microenvironmental‐sensitive platform to load recombinant human type III collagen (rhCol III) and PDA@Ag NPs. Upon exposure to the microenvironment, the hydrogel structure was fragmented, releasing rhCol III and PDA@Ag NPs. rhCol III facilitated the activation of fibroblasts and endothelial cells, while PDA@Ag NPs presented superior anti‐inflammatory and antibacterial effects. Such a hydrogel was excellent for satisfying the substance needs of various stages of wound healing.^[^ ^141^ ^]^ In addition to collagen, proline, a procollagen component was also implanted into a nanofiber network to stimulate fibroblast proliferation.^[^ ^109b^ ^]^

For the *Condition III*, gene therapy based on MMP‐assisted small interfering RNA (siRNA) has been extensively studied to silence its expression, and a functional nanosystem can effectively reduce the degradation of siRNA.^[^ ^16^, ^142^ ^]^ Accordingly, the MMP‐2 siRNA was electrostatically anchored into linear polyethyleneimine (LPEI) which was conjugated on the surface of the nanofiber mesh. After treatment with this mesh, the expression level of MMP‐2 was significantly reduced within 7 d and, compared with the untreated group, the wound area exhibited a 3.5‐fold deposition of new collagen fibers.^[^ ^18i^ ^]^

### 4.7 Promotion of Re‐epithelialization

Similarly, re‐epithelialization is coupled with the activation of keratinocytes (migration and proliferation). To achieve re‐epithelization, the most common strategy is the exogenous delivery of cytokines to facilitate the activation of keratinocytes.^[^ ^85^, ^143^ ^]^ For instance, Yoon et al. prepared a chemokine‐loaded hydrogel that effectively recruited cells to the wound, and further promoted angiogenesis, collagen deposition, and re‐epithelialization.^[^ ^85a^ ^]^ Furthermore, lipid NP implanted with recombinant human EGF was reported to possess the ability to stimulate fibroblast and keratinocyte proliferation; then, the epithelialization promotion evaluation on animal models (using criteria established by Sinha et al.,^[^ ^144^ ^]^) demonstrated that wounds in the nanosystems treatment group showed a more pronounced healing effect.^[^ ^145^ ^]^

In addition to direct delivery of cytokines, other “cargoes” such as neutralizing antibodies,^[^ ^146^ ^]^ engineered exosomes,^[^ ^147^ ^]^ and stem cells^[^ ^148^ ^]^ can also enhance the viability of keratinocytes. Here, the Flightless I (Flii) neutralizing antibody was loaded into porous silicon NPs to downregulate Flii overexpressed in DFU. As expected, the nanodelivery system reduced the degradation of Flii‐neutralizing antibodies and effectively promoted keratinocyte activation by reducing Flii expression, resulting in an accelerated wound healing rate in diabetic mice compared to the control group (≈18% faster).^[^ ^146^ ^]^ In another strategy, the miR‐31‐5p (low expression in DFU) mimic was infiltrated into exosomes. MiR‐31‐5p, a versatile factor regulating angiogenesis, fibrogenesis, and re‐epithelialization, is associated with its capability to oppress the factor‐inhibiting HIF‐1 and epithelial membrane protein‐1 expression, which in turn facilitate VEGF expression and keratinocyte activation, respectively.^[^ ^147^ ^]^

In general, nanotechnology has greatly lifted the limitations of biologically active components (cells, antibodies, cytokines, etc.) for therapeutic purposes, thus displaying pronounced proangiogenic, pro‐ECM remodeling, and/or re‐epithelialization effects. However, the stability of the active ingredients in nanosystems also requires careful assessment, as such systems construction involves organic reagents presence. As well, direct comparisons with clinically relevant dressings or available products are absent in many studies, which needs to be performed to tailor clinical scenarios.

Apart from the above strategies, some nanomaterials themselves possess pro‐epithelialization functions, such as MOF^[^ ^149^ ^]^ and nanofibers.^[^ ^150^ ^]^ As mentioned earlier, Cu features various biomedical effects and therefore Cu ions released from Cu‐MOF could promote angiogenesis, spur collagen formation, and induce epithelialization.^[^ ^149^ ^]^

Finally, some gas‐based therapies, such as H_2_S^[^ ^118c^ ^]^ and O_2_, also promote re‐epithelialization. See Sections 4.5 and 4.8.

### 4.8 Regulation of Hypoxia

**Figure 7:**
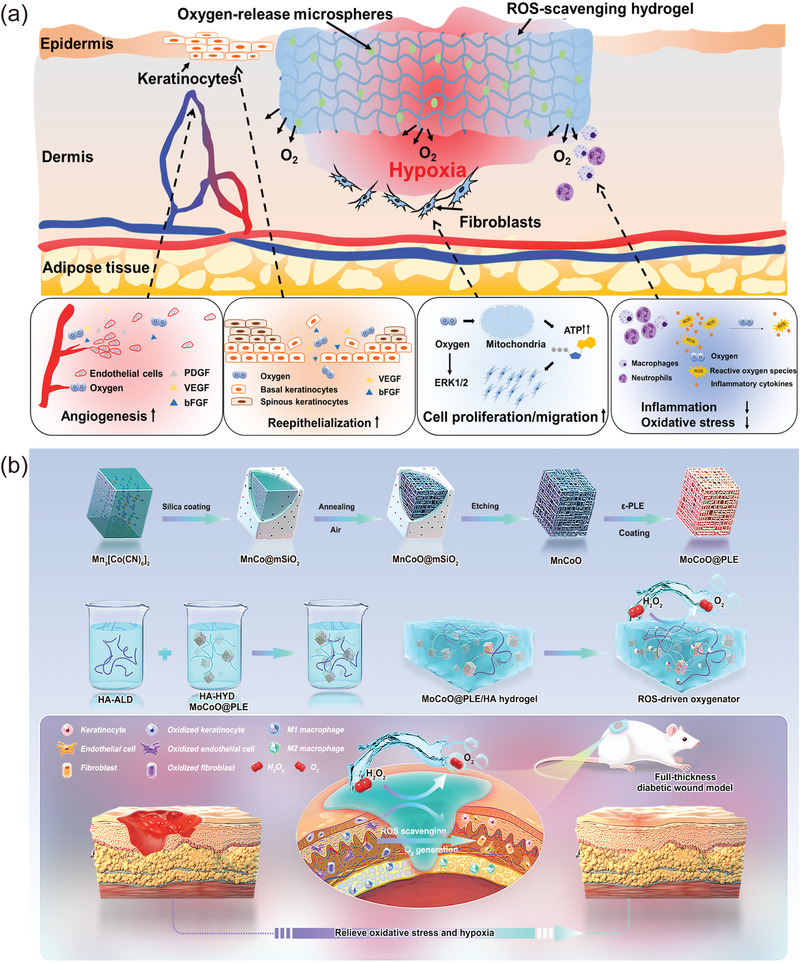
a) Effect of oxygen release on wound microenvironment and its mechanism of accelerated healing. Reproduced with permission.^[^ ^53a^ ^]^ Copyright 2021, American Association for the Advancement of Science. b) Schematic illustration indicated the synthesis process of nanozyme‐reinforced self‐protecting hydrogels as ROS‐driven oxygenerator and the application for enhancing diabetic wound healing. Reproduced with permission.^[^ ^157^ ^]^ Copyright 2022, Wiley‐VCH.

As previously mentioned, a hypoxic microenvironment develops in DFU due to unbalanced oxygen supply and demand. Studies have shown that the administration of local oxygen to the wound remodels the wound microenvironment and regulates multiple processes of wound healing, such as anti‐inflammation and promotion of angiogenesis (**Figure** **7**a).^[^ ^48^, ^53^, ^151^ ^]^

Considering the necessity of oxygen, hyperbaric oxygen therapy and topical gaseous oxygen are applied clinically to promote wound oxygenation. However, poor oxygen level maintenance and wound permeability prevent such therapies from achieving satisfactory results.^[^ ^53^, ^152^ ^]^ Therefore, various nanosystems have been designed to surmount these conundrums by enhancing skin permeability and sustaining oxygen release, such as nanosystems based on hemoglobin,^[^ ^153^ ^]^ calcium peroxide (CaO_2_),^[^ ^125a^ ^]^ H_2_O_2_,^[^ ^67^, ^154^ ^]^ nanoperfluorocarbon,^[^ ^155^ ^]^ and oxygen‐producing polymer nanofibers.^[^ ^156^ ^]^ Specifically, the hemoglobin with oxygen release and binding capability was implanted into microneedles loaded with BP quantum dots (BP QDs) for wound healing. In this system, microneedles were able to penetrate the epidermal layer to overcome the skin barrier. Furthermore, under laser irradiation, the photothermal capacity of BP QDs was utilized to increase the local temperature of the wound, further promoting the oxygen release capacity of hemoglobin rather than binding capacity, and ultimately achieving controlled oxygen release lasting for 24 h.^[^ ^153^ ^]^ In addition to hemoglobin, the utilization of antioxidant nanoenzymes (e.g., CeO_2_, MnO_2_, mesoporous manganese cobalt oxide [MnCoO]) to catalyze oxygen production accompanying ROS scavenging has also been investigated as a promising pathway for regulating the immune microenvironment.^[^ ^157^ ^]^ For instance, Zhang et al. developed hydrogels containing MnO_2_ nanoenzymes, which effectively modulated the inflammatory and hypoxia microenvironment by catalyzing the decomposition of H_2_O_2_ into oxygen at the wound.^[^ ^154^ ^]^ Another typical illustration reported by Wang et al. is a CAT‐mimic nanoenzyme (*ε*‐polylysine coated MnCoO) immobilized hydrogel with ROS scavenging and oxygen correcting capabilities. Such a nanocatalytic system with continuous catalytic capacity and prolonged durability effectively enhances healing (Figure 7b).^[^ ^157^ ^]^ The aforementioned studies suggest that ROS scavenging and hypoxia correcting materials to achieve catalytic therapy by nanoenzymes may bring a breakthrough for DFU nanomedicine.

Apart from direct oxygen release, growth factors are also clinically administered to improve wound oxygenation, but their applications are limited. Strategies to address growth factor delivery conundrums by nanosystems are described in Section 4.5.

Admittedly, nanosystems remove some of the restrictions of existing oxygen therapy, but there are still some issues that need to be addressed to unleash their maximum potential. First, more trials are necessary to determine its efficiency in wound oxygenation due to the effectiveness of growth factor therapy in wound healing is not well established (only PDGF is approved). In addition, it is generally ideal to maintain subcutaneous oxygen tension at 40 mmHg.^[^ ^158^ ^]^ However, many studies do not monitor wound oxygen levels. Finally, the safety, controllability, and stability of oxygen release from nanosystems should also be investigated in depth to accelerate clinical translation.

### 4.9 Others

Except for above mentioned, nanosystems based on neural repair, diagnosis, or the integration of diagnosis and treatment have also been explored. For example, Cui et al. prepared a smart dressing that could be used to treat DFU and detect the level of H_2_O_2_ in wounds—first modifying the luminescent porous silicon (PSi) material with graphene quantum dots (GQDs@PSi), then loading insulin and EGF into GQDs@PSi (BiPEP‐GQDs@PSi), and finally incorporating it in a chitosan film. Diagnostically, the intrinsic fluorescence of GQDs@PSi would shift if the ROS level changed, so that it could be used for feedback of oxidative stress level at the wound; therapeutically, the two peptides embedded in the material effectively could promote wound healing via lowering blood glucose and facilitating related cell activation.^[^ ^159^ ^]^ Noticeably, although such smart dressings show great potential, effective clinical diagnostic systems are still relatively lacking to monitor the wound healing process. To this end, much more research needs to be done to develop more efficient, convenient, and accurate nanosystems to open the “black box” of the ulcer healing process so that doctors can easily determine the disease status.

## 5 Conclusions and Prospects

**Figure 8:**
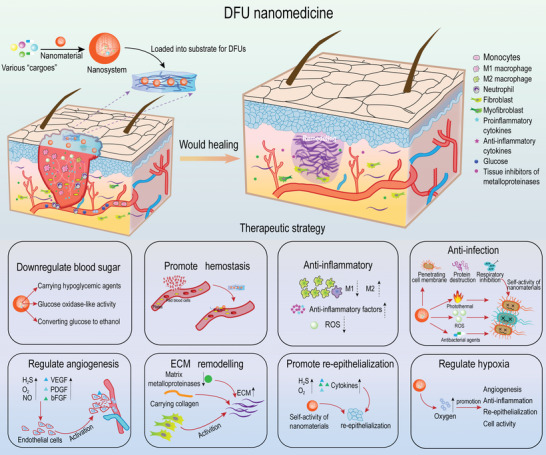
Therapeutic strategies of DFU nanomedicines, such as downregulating blood sugar, hemostasis, and anti‐inflammatory. Nanosystems usually perform unitary or multiple functions for DFU therapy with the assistance of macroscopic substrates such as hydrogels (most predominantly), fibers, and microneedles.

Considering the severity and undesirable prognosis of DFU, a number of DFU nanomedicines have been continuously explored. Based on the wound healing process and the complex microenvironment of DFU, researchers have designed nanosystems in two main ways: 1) Utilizing the chemical intrinsic activities of nanomaterials; and 2) delivering active ingredients, such as antibiotics, anti‐inflammatory drugs, hypoglycemic drugs, enzymes, growth factor stem cells, exosomes, or genes by nanomaterials based carriers. Such constructed nanosystems can achieve “glucose‐lowering plus antibacterial” or “anti‐inflammatory plus proangiogenic” roles, or other additional functions, and ultimately contribute to wound healing (**Figure** **8**). Undeniably, these strategies offer innovative approaches to the treatment of diseases. However, more extensive researches are imperative to translate these strategies to the clinic: 1) bio‐safety: currently the physicochemical properties and the reproducible preparations of DFU‐associated nanomaterials are still unsatisfactory from the viewpoint of clinic application; (2) the biodistribution, degradation process, and biological effects, especially the long‐term effect of nanomedicine in the body, are still not completely clear, and the highly efficient targeting of nanomedicine at specific sites requires further excavation; 3) more in vivo studies on large mammals, rather than the current mice or rats mostly used in literature, are desired; 4) simpler, more controllable, and reproducible agents are developed to facilitate translation; and 5) more specific and/or convenient administration methods are to be developed for enhanced efficacy and clinic handiness.

In the future development of DFU nanomedicines, in addition to the above challenges which need better solutions, the following advances in the future can be envisaged: 1) the drug delivery efficiency and the self‐activity of nanosystems should be further improved to enhance the bioavailability of nanomedicines; 2) more effective “multifunctional nanosystems” will be developed based on the complex microenvironment of DFU; 3) diagnosis and treatments are to be integrated into one nanosystem; 4) nanotechnology‐based gene therapy, cell therapy, growth factor therapy, etc., are to be explored and established based on the effectiveness of these factors for wound healing; 5) currently, most nanomedicines have only been explored phenotypically (e.g., anti‐inflammatory, antibacterial, modulation of signaling pathways); however, research on the precise mechanisms (e.g., exact sites of action, targets of molecular interactions) is still lacking. In‐depth mechanism studies are highly desired for rational guidance of nanomaterial design and clinical administration; 6) nanomedicines developed in other fields, such as tumor therapeutics, will be used for references for DFU treatment, according to the similar or comparable microenvironments between malignant tumors and DFU, such as high oxidative stresses and upregulated expressions of proteases. For example, the concept of “nanocatalytic medicine,” which has been proposed in recent years mainly for tumor treatment, may also be therapeutically effective in the field of DFU therapeutics. Specifically, a number of nanocatalysts mimic the activities of anti‐inflammatory enzymes such as catalase, superoxide dismutase, and glutathione peroxidase to reduce ROS levels, and could thus be introduced to reshape the microenvironment of DFU; 7) traditional Chinese medicine (such as *Angelica dahurica*, *Radix astragali*, and *Radix rehmanniae*) is highly effective in treating DFU in some cases, which should be further probed in detail. Also, they may be processed into nanoscale for better efficacy in DFU treatment; and 8) in addition, high recurrence rates are one of the keys to the obstacles in DFU healing. Thus, designing anti‐relapse nanosystems has great potential for DFU treatment.

Taken together, multifunctional nanosystems have brought unprecedented advances in DFU treatment and the fusion of multiple research techniques. Despite there are obstacles in the practical application of DFU nanomedicines, it is indisputable that clinical DFU nanomedicines will be developed with the concerted efforts of scientists in various fields.

## Conflict of Interest

The authors declare no conflict of interest.
